# Genome-Wide Association Study Reveals the QTLs for Seed Storability in World Rice Core Collections

**DOI:** 10.3390/plants10040812

**Published:** 2021-04-20

**Authors:** Fangxi Wu, Xi Luo, Lingqiang Wang, Yidong Wei, Jianguo Li, Huaan Xie, Jianfu Zhang, Guosheng Xie

**Affiliations:** 1 Rice Research Institute, Fujian Academy of Agricultural Sciences, Fuzhou 350019, China; wufangxi@faas.cn (F.W.); luoxi@faas.cn (X.L.); weiyidong@faas.cn (Y.W.); huaanxie@faas.cn (H.X.); 2 MOA Key Laboratory of Crop Ecophysiology and Farming System in the Middle Reaches of the Yangtze River, College of Plant Science and Technology, Huazhong Agricultural University, Wuhan 430070, China; 3 State Key Laboratory for Conservation and Utilization of Subtropical Agro-Bioresources, College of Agriculture, Guangxi University, Nanning 530004, China; lqwang@mail.hzau.edu.cn (L.W.); ooozil0325@gmail.com (J.L.); 4 College of Plant Science and Technology, Huazhong Agricultural University, Wuhan 430070, China

**Keywords:** rice (*Oryza sativa* L.), seed storability, GWAS, QTL, SNP

## Abstract

Seed storability is a main agronomically important trait to assure storage safety of grain and seeds in rice. Although many quantitative trait loci (QTLs) and associated genes for rice seed storability have been identified, the detailed genetic mechanisms of seed storability remain unclear in rice. In this study, a genome-wide association study (GWAS) was performed in 456 diverse rice core collections from the 3K rice genome. We discovered the new nine QTLs designated as *qSS1-1*, *qSS1-2*, *qSS2-1*, *qSS3-1*, *qSS5-1*, *qSS5-2*, *qSS7-1*, *qSS8-1*, and *qSS11-1*. According to the analysis of the new nine QTLs, our results could well explain the reason why seed storability of *indica* subspecies was superior to *japonica* subspecies in rice. Among them, *qSS1-2* and *qSS8-1* were potentially co-localized with a known associated *qSS1*/*OsGH3-2* and *OsPIMT1*, respectively. Our results also suggest that pyramiding breeding of superior alleles of these associated genes will lead to new varieties with improved seed storability in the future.

## 1. Introduction

Rice (*Oryza sativa* L.) is one of the staple crops worldwide. In recent years, nearly 3% of the average annual rice grain, namely 15 billion kg, has been lost due to seed aging during storage [1]. Seed deterioration after harvest is a serious problem for rice production in Asia, especially for hybrid rice in the Southern area [2]. The loss of seed viability due to seed deterioration has been a great challenge to the crop production industry [3]. Therefore, the improvement of seed storability is of great significance to assurance storage safety of grain and seeds in rice.

Seed storability is defined as the longevity of seeds after storage. It is an important agronomic trait for the preservation of seed fitness after harvest [4]. Generally, seed storability is affected by genetic and environmental factors during plant growth, seed maturation, and post-harvest. Improvement of seed storability environment spends a large amount of manpower and material costs, and it is also not economic. Nevertheless, the improvement of genetic factors for rice seed storability is very effective through genetic breeding. Recent researches have demonstrated that seed storability varies greatly among different rice accessions, and seed storability of *indica* subspecies is better than that of *japonica* subspecies [5,6,7], suggesting that there are different genetic mechanism and interaction network for seed storability between *indica* and *japonica* subspecies.

During the past decade, through the quantitative trait loci (QTLs) analysis and association mapping approaches, more than 70 QTLs for seed storability in rice have been identified under natural storage or artificial aging conditions. After keeping seeds for 1, 2, and 3 years, Sasaki detected four QTL (*RC7*, *RC9-1*, *RC9-2*, and *RC9-3*) for seed longevity in 191 Recombinant Inbred Lines (RILs) derived from Milyang 23/Akihikari [8]. Jiang found seven QTLs associated with seed storability using two sets of RILs from Milyang23/Tong 88-7 and Dasanbyeo/TR22183 [9]. Li identified six QTLs affecting seed storability on chromosomes 2, 3, 4, 6, 9, and 11 in a backcross population of Koshihikari/Kasalath after natural aging for 32 and 48 months [10]. On the other hand, using a backcross population of Nipponbare/Kasalath, Miura identified three QTLs *qLG-2*, *qLG-4*, and *qLG-9* related to seed longevity on chromosome 2, 4, and 9 through the artificial aging method [11]. Zeng detected three QTLs *qLS-9*, *qLS-11*, and *qLS-12* for seed storability to explain 35.4% of the genetic variation using doubled-haploid population derived from the cross ZYQ8/JX17 [12]. Xue isolated three QTLs associated with seed storability on chromosomes 1, 3, and 9 using RILs derived from the cross IR24/Asominori [13]. Lin identified seven QTLs on chromosomes 1, 2, 5, 6, and 9 by using two backcross-inbred populations with N22 as a common parent after natural, artificial, and combined aging treatments [14]. Hang used seeds of backcross-inbred lines treated under natural and artificial aging storage conditions and identified 13 QTLs for seed storability on chromosomes 1, 2, 3, 4, 5, 7, 11, and 12. Among them, two QTLs were detected in both conditions, four and seven QTLs were detected either in natural or artificial aging treatments [4]. Li used a doubled haploid population during natural storage or artificial aging and identified 19 QTLs on nine chromosomes with phenotypic variations ranged from 2.1% to 22.7% [15]. These results above deepen the understanding of genetic mechanisms of seed storability and will be useful for breeding new rice varieties with high seed storability.

Nevertheless, only a few QTLs have been finely mapped, such as *qSS-9* or *qGP-9*, *qSS1*, and *qSS3.1* [14,15,16]. Besides, several genes that influence seed storability have been cloned, such as aldehyde dehydrogenase *OsALDH7* [17], lipoxygenases *OsLOX2* and *LOX3* [1,18], L-isoaspartylmethyltransferases *OsPIMT1* and *PIMT2* [19,20], Metallothionein 2b (*OsMT2b*) [21]. Just recently, Yuan identified a seed storability-associated gene *OsGH3-2* by a genome-wide association study in rice germplasms with linkage mapping in chromosome substitution segment lines after natural storage and artificial aging treatments. Moreover, transgenic experiments demonstrated that *OsGH3-2* acted as a negative regulator of seed storability by modulating the abscisic acid (ABA) pathway [22]. On the other hand, Lee firstly identified eight major loci associated with seed longevity by GWAS of a panel of 299 *indica* accessions, and the favorable haplotypes on chromosomes 1, 3, 4, 9, and 11. Moreover, they selected a priori candidate genes involved in DNA repair and transcription, sugar metabolism, reactive oxygen species (ROS) scavenging, and embryonic and root development processes. Overall, these findings shed light on the complex mechanisms of seed storability and will facilitate the improvement of seed vigor by genomic breeding in rice.

Therefore, to identify the quantitative trait loci (QTLs) and candidate genes associated with seed storability in rice, we applied a GAPIT (Genomic Association and Predication Integrated Tool) method with MLM in a GWAS of 456 diverse rice core collections from the 3K rice genome. Accordingly, nine main QTLs with major effects were identified on chromosomes 1, 2, 3, 5, 7, 8, and 11. We also explain that why *indica* subspecies had superior seed storability to *japonica* subspecies. Our results indicate that pyramiding of superior alleles of these genes for seed storability will lead to new varieties with improved seed longevity and storage in the future.

## 2. Results

### 2.1. Population Structure and Phenotypic Evaluation of Seed Storability in Rice

**Figure 1:**
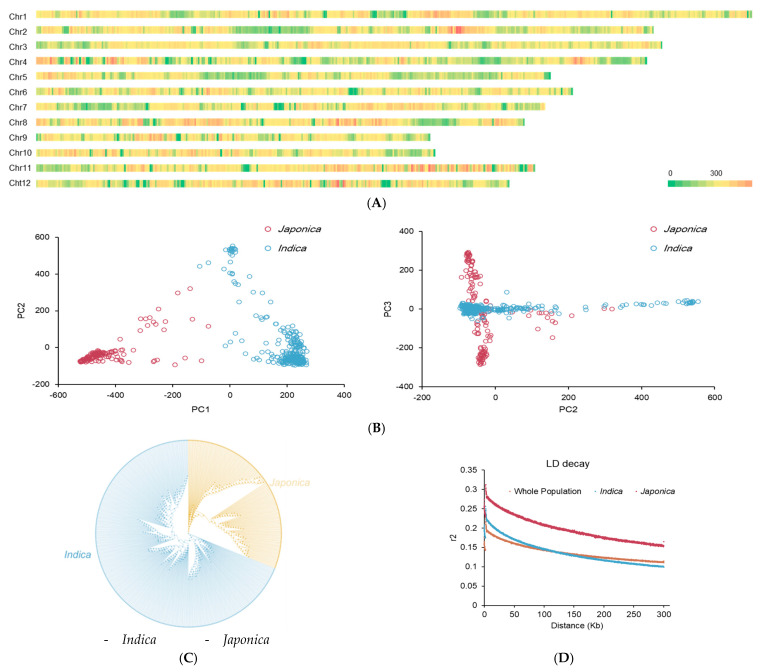
The distribution of SNPs, analysis of population structure, and LD decay of 456 rice accessions. (**A**) The distribution of SNPs in the rice genome analysis of population structure. (**B**) principal component, the red indicates the *indica* subspecies, while the purple indicates the *japonica* subspecies. (**C**) Phylogenetic tree: the blue indicates the *indica* subspecies, while the yellow indicates the *japonica* subspecies. (**D**) LD (linkage disequilibrium) decay of 456 rice accessions in the whole population, *indica* subspecies, and *japonica* subspecies, subspecies.

We chose 584,145 SNPs from the 3K rice genome project 1M GWAS SNP dataset from http://snp-seek.irri.org/_download.zul (accessed 25 December 2020). These SNPs evenly distribute on twelve chromosomes, and their average density is 1.3 SNP/Kb in our population (Figure 1A). We performed the population structure analysis based on 584,145 SNPs in the whole population by using the ADMIXTURE software (Figure 1B). The results showed that there were two subpopulations by using the first three principal components. The phylogenetic tree analysis also showed that all 456 rice accessions were divided into *indica* subspecies (313 accessions, 68.64%) and *japonica* subspecies (143 accessions, 31.36%) (Figure 1C and Supplementary Table S1). The decay distance of LD with the physical distance was at 270, 150, and 280 kb (r^2^ =0.2), in the whole population, *indica* subspecies, and *japonica* subspecies (Figure 1D), respectively.

**Figure 2:**
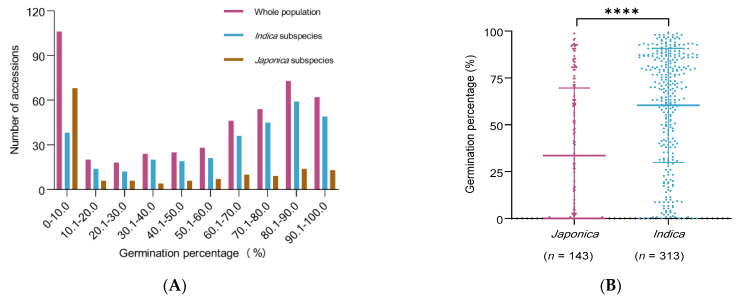
The difference phenotype of 456 rice accessions. (**A**) Distribution and variations of seed germination percentage at temperature 42 °C and relative humidity 88% for 20 days in the whole population, *indica* subspecies, and *japonica* subspecies. (**B**) Scatter dot plot of seed germination percentage in *indica* subspecies and *japonica* subspecies. **** denote significant differences in mean phenotypic between superior and inferior alleles at *p* > 0.0001.

Phenotypic variations of seed storability among the 456 rice accessions were evaluated at the laboratory in Fuzhou during the winter season of 2020. Large variations in seed germination percentage were observed in the whole population under temperature 42 °C and relative humidity 88% for 20 days, ranging from 0 to 99.3%, with an average of 52.0% (Figure 2A). The seed germination percentage distribution in 456 accessions was continuous, with more in the low germination percentage side; the seed germination percentage of the 106 accessions (23.2%) was less than 10.1% while the seed germination percentage of 19 accessions (4.0%) was in 20.1~30.0%. The results showed that the difference was significant for the seed storability of these core collections.

Large variations of seed germination percentage were also observed, and percentage distribution was also continuous in both *indica* and *japonica* subspecies under temperature 42 °C and relative humidity 88% for 20 days. The results also showed that the difference was significant for the seed storability in these core collections both *indica* and *japonica* subspecies.

The comparisons of seed germination percentages between different subspecies revealed that the seed germination percentage of *indica* subspecies was significantly higher than that of *japonica*, and the average germination percentage was 60.4% in *indica* subspecies while it was 33.5% in *japonica* subspecies. Besides, the germination percentage of the 38 accessions (12.1%) was less than 10.1% in *indica* subspecies while it was 68 accessions (47.6%) in *japonica* subspecies (Figure 2A,B). T-test of seed germination percentage between different subspecies showed that t value equaled to 8.24, and *p*-value was less than 0.0001, and level of significance reached ****. The results clearly showed that the seed storability was significantly different with *indica* and *japonica* subspecies, and that of *indica* subspecies was better than that of *japonica* subspecies.

### 2.2. Identification of Nine New QTLs for Seed Storability by GWAS in Rice

We used the PLINK program (version 1.9) to obtain a subset of 584,145 SNPs with a minor allele frequency > 5% and a missing data ratio <0.2 for association analyses in the population. Because the seed storability was significantly different with *indica* and *japonica* subspecies, we performed the mixed linear model (MLM) with the first three principal components as covariates by using GAPIT (version 2) to identify the association signals in the whole population, *indica* subspecies, and *japonica* subspecies, respectively.

Because using the false discovery rate (FDR) as the threshold value is very strict, we selected *p* < 0.0001 as the threshold value as previously reported [23,24,25,26]. We considered that there is a QTL in the SNP peak place. Besides, in the SNP peak place, there should be three or more three consecutive significant SNPs (*p* < 0.0001) in adjacent significant SNPs with distances less than 270, 150, and 280 kb in the whole population, *indica* subgroup, and *japonica* subgroup, respectively.

**Figure 3:**
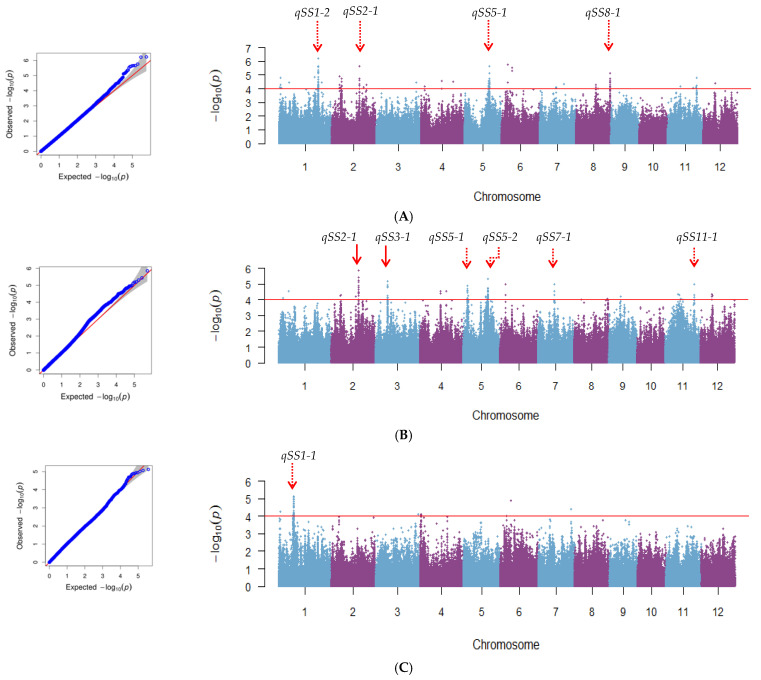
Quantile-quantile (Q-Q) and Manhattan plots of GWAS for rice seed storability in the whole population (**A**), *indica* subspecies (**B**), and *japonica* subspecies (**C**), respectively.

**Figure 4:**
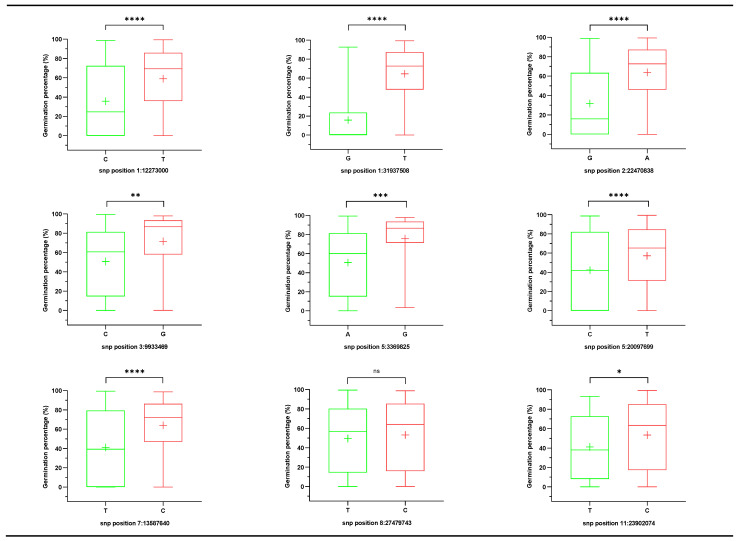
The box plots showing the phenotypic distribution at the nine superior and inferior alleles of lead SNPs in the world core collection. The plus shows the mean; the middle line shows the median, and the box shows the range of the 25th to 75th percentiles of the total data and the whiskers show the interquartile range and the outliers. ns, *, **, *** and **** denote significant differences in mean phenotypic between superior and inferior alleles at *p* > 0.05, *p* < 0.05, 0.01, 0.001, and 0.0001, respectively.

Therefore, according to the above method, we identified 107, 129, and 26 SNPs for seed storability at -log (P) significance levels of 4 in the whole population, *indica* subspecies, and *japonica* subspecies, respectively, using MLM (Figure 3). As result, 4, 6, and 1 QTL was identified by GWAS using MLM in the whole population, *indica* subspecies, and *japonica* subspecies, respectively (Figure 3, Table 1). They were distributed on chromosomes 1, 2, 3, 5, 7, 8, 11 and designated as *qSS1-1*, *qSS1-2*, *qSS2-1*, *qSS3-1*, *qSS5-1*, *qSS5-2*, *qSS7-1*, *qSS8-1*, and *qSS11-1*. Moreover, *qSS2-1* and *qSS5-1* had been detected in both the whole population and *indica* subspecies. Accessions in world core collection with different alleles for the nine SNPs appeared distinct variance of phenotypes (Figure 4). Besides, the effect value of the new nine SNPs is 6.36–17.51%, 0.46–32.48%, 0.33–22.97% in the whole population, *indica* subspecies, and *japonica* subspecies, respectively (Table 2).

Among these QTLs, *qSS1-2* and *qSS8-1* were potentially co-localized with known associated *qSS1*/*OsGH3-2* and *OsPIMT1* respectively. The lead SNP of *qSS1-2* was 290 kb away from *OsGH3-2* (*OsGRETCHENHAGEN3-2*, Os01g0764800) [22], which was reported to be related to seed storability in rice. Its mechanism of action is that the inactivation of indole-3-acetic acid leads to increase seed storability in rice. The lead SNP of *qSS8-1* was 398 kb away from *OsPIMT1* (Os08g0557000) [19], which was reported that *OsPIMT1* probably repair detrimental isoAsp-containing proteins that over accumulate in aging rice seed embryos.

### 2.3. Pyramiding Analysis of These QTLs for Rice Seed Storability

**Figure 5:**
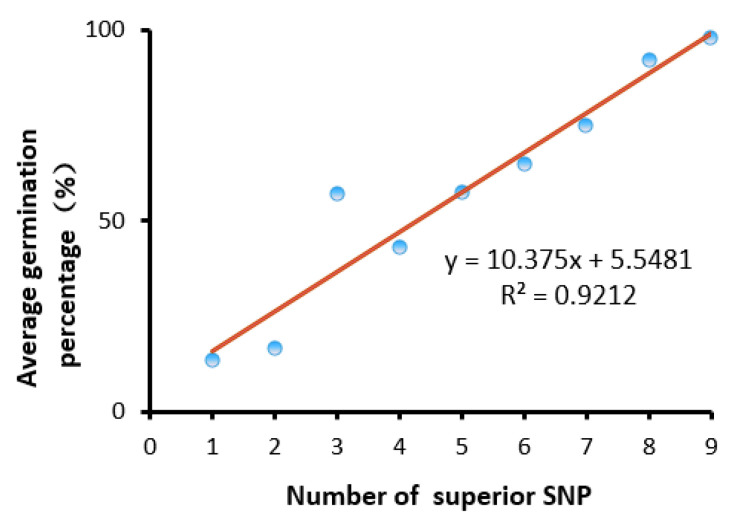
The average germination percentage of pyramiding QTL superior alleles in the whole population.

Then, we examined the correlation of the number of QTL superior alleles with average germination percentage and thus found that the average germination percentage was increased with the number of QTL superior alleles (Figure 5) in the whole population. Then, we analyzed the main haplotypes of the nine QTLs for seed storability in both subspecies (Table 3), eight main haplotypes in *indica* subspecies were discovered, and the average germination percentage increased with the number of QTL superior alleles. On the other hand, there were six main haplotypes in *japonica* subspecies. As unexpected, the superior haplotype with *qSS1-2*, *qSS8-1*, and *qSS11-1* displayed a higher average germination percentage than the superior haplotype with *qSS1-1, qSS1-2*, *qSS8-1*, and *qSS11-1* in *japonica* subspecies. These results indicated that pyramiding of QTL superior alleles of these seed storability-associated genes would be essential for breeding a new rice variety with improved storability.

## 3. Discussion

### 3.1. Seed Storability Variations between Indica and Japonica Subspecies in Rice

Seed storability is an important agronomic trait for the conservation of seed resources and quality that determines the longevity of seeds after storage, Therefore, a reliable assay is essential to accurately phenotype the response to seed storability. Because natural storage is not economic and small scale, the artificial aging treatment has been used as an alternative to analyzing this seed property more efficiently. High seed moisture content and high-temperature treatments can artificially accelerate seed aging. In this way, seed storability of rice germplasm has been evaluated based on the seed germination test at normal conditions [13]. On the other hand, there were significant differences in storage properties among rice germplasm and accessions from different geographical regions [5,12]. Kameswara and Jackson compared the seed storability of 16 Asian and one Africa rice cultivars and found that the order of seed storability was the following: *indica* > *javanica* > *japonica* [27]. Lee detected a great difference in seed storability of 299 *indica* rice accessions [7].

We evaluated the seed storability of 456 rice core collections under the artificial aging treatment of temperature 42 °C and relative humidity 88% for 20 days and also found that the seed storability was significantly different with *indica* and *japonica* subspecies and that of *indica* subspecies was better than that of *japonica* subspecies.

**Figure 6:**
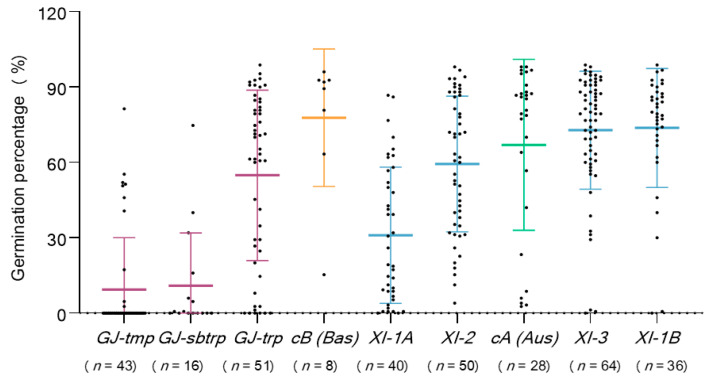
Scatter dot plot of seed germination percentage at temperature 42 °C and relative humidity 88% for 20 days in different subgroups.

In this study, we collected 456 rice core collections of 47 different countries that included four XI clusters: XI-1A from East Asia, XI-1B of modern varieties of diverse origins, XI-2 from South Asia, and XI-3 from Southeast Asia; Three GJ clusters: GJ-tmp from East Asian temperate primarily, GJ-sbtrp from Southeast Asian subtropical and GJ-trp from Southeast Asian tropical; and single groups of cA and cB accessions for the mostly South Asian [28]. In more detail, among the five *indica* subgroups, the order of seed storability was XI-1B > XI-3 >Aus > XI-2 > XI-1A. Among the four *japonica* subgroups, the order of seed storability was Bas > GJ-trp> GJ-sutrp > GJ-tmp (Figure 6). These new results indicate that the origin and natural habitat greatly affect the seed storability of rice germplasm. Rice germplasms are more storable in tropical and subtropical origins than that in temperate origins; it will be easier to obtain the seed storable germplasm of rice in these tropical and subtropical regions.

### 3.2. GWAS for Seed Storability by Using High-Throughput Genotyping in Rice

As described above, more than 70 quantitative trait loci (QTLs) controlling seed storability have been identified by genetic segregating populations under either natural or artificial aging in rice [4,8,9,10,11,12,13,29,30]. In this study, we identified nine new QTLs for rice seed storability by GWAS analysis. They were distributed on chromosomes 1, 2, 3, 5, 7, 8, 11 and designated as *qSS1-1*, *qSS1-2*, *qSS2-1*, *qSS3-1*, *qSS5-1*, *qSS5-2*, *qSS7-1*, *qSS8-1*, and *qSS11-1*. In addition, the effect value of the new nine SNPs is 6.36%–17.51%, 0.46%–32.48%, and 0.33%–22.97% in the whole population, *indica* subspecies, and *japonica* subspecies, respectively. In these QTLs detected, only *qSS1-2* and *qSS8-1* were potentially co-localized with known associated qSS1/OsGH3-2 and OsPIMT1, respectively, which was related to rice seed storability [16,19,22]. The results illustrated that seed storability is a complex quantitative strait controlled by polygenes in rice.

In our GWAS, we had identified a range of genes with allelic variation among a large number of diverse accessions. Interestingly, there is a typically higher resolution of QTLs, allowing the direct identification of candidate genes without the need for further fine mapping [31]. Although a GWAS panel could span different variety groups, haplotypes are often subpopulation-specific [32]. Consequently, restricting a panel to a single variety group increases the power of QTL detection for genes that are polymorphic only within that variety group [33,34].

### 3.3. Exposing the Difference of the Two Subspecies for Rice Seed Storability by Analysis Superior Allele Frequency of the QTLs

**Figure 7:**
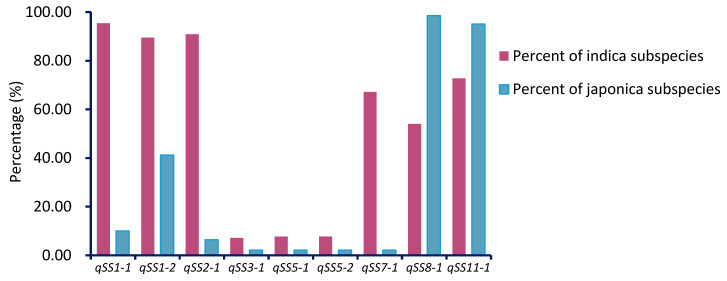
Comparison of the frequency of these QTLs superior alleles in *indica* and *japonica* subspecies.

We divided the population into two groups according to allelic genotypes. As a result, the superior allele frequency of *qSS1-1*, *qSS1-2*, *qSS2-1*, *qSS3-1*, *qSS5-1*, *qSS5-2*, and *qSS7-1* was higher in *indica* than *japonica* subspecies. However, only the superior allele frequency of *qSS8-1*, *qSS11-1* was higher in *japonica* than *indica* subspecies (Figure 7). The results indicated these superior alleles for the seed storability more widely distribute in *indica* than *japonica* subspecies. This could well explain the reason why the seed storability of *indica* subspecies was superior to *japonica* subspecies in rice.

### 3.4. New SNPs Participate in the Seed Storability in Rice

Seed deterioration after harvest is a serious problem for rice production in Asia, especially for hybrid rice in the Southern area [2]. The loss of seed viability due to seed deterioration has been a great challenge to the crop production industry [3]. Recent researches have demonstrated that seed storability varies greatly among different rice accessions. In this paper, a genome-wide association study (GWAS) was performed in 456 diverse rice core collections from the 3K rice genome. We discovered the nine new SNPs about rice seed storability and found that the average germination percentage was increased with the number of these superior *SNPs*. Therefore, the pyramiding of these new *SNPs* would be essential for breeding a new rice variety with improved storability.

## 4. Materials and Methods

### 4.1. Plant Materials

The rice population comprising 456 cultivated rice accessions was collected from the 3K Rice Genome (3K-RG) and obtained from the Institute of Crop Sciences, Chinese Academy of Agricultural Sciences, Beijing, China. These accessions were core collections from 47 counties representing major rice-growing regions of the world (Table S1). All seeds used for artificial aging treatment (AAT) experiments in this study were harvested from Fujian rice Breeding Base in Sanya City, Hainan Province, China, 2020.

### 4.2. Artificial Aging Treatment

Newly harvested rice seeds were stored at room temperature for 6 months to break dormancy. The artificial aging treatment used in this study was as described by Zeng with some modifications [35]. To accelerate aging, these seeds were stored at temperature 42 °C and relative humidity 88% for 20 days in a closed desiccator (BINDER GmbH, Germany) with a thermostatic moisture regulator. The fifty healthy and filled seeds were treated from each sample with three replicates.

### 4.3. Seed Germination Test

The treated seeds were sown on two layers of filter paper [35] and germinated in an incubator at temperature 30 °C/relative humidity 75% with 14 h of light per day for 12 days. The germination percentage was measured as the number of germinated seeds divided by total seeds after 12 days.

### 4.4. Genotyping and GWAS for Rice Seed Storability

The 456 rice accessions analyzed in this study were from the 3K rice genome project [36]. The 3K rice genome project 1M GWAS SNP dataset from the Rice-Seek Database was downloaded from http://snp-seek.irri.org/_download.zul (accessed 25 December 2020). We used the PLINK program (version 1.9) [37] to obtain a subset of 584,145 SNPs with a minor allele frequency >5% and a missing data ratio < 0.2 for association analyses in the population. The population structure was analyzed by using ADMIXTURE software [38]. The phylogenetic tree was constructed according to the method of Saitou M [39].

The decay distance of LD (linkage disequilibrium) in the whole population, *indica* subspecies, and *japonica* subspecies was analyzed by software PopLDdecay, respectively [40].

A mixed linear model (MLM) was performed by using the SNP set and default settings GAPIT 2.0 (Genomic Association and Predication Integrated Tool) in the whole population, *indica* subspecies, and *japonica* subspecies, respectively [41]. The first three PC were used to construct the PC matrix and kinship matrix analyses. Because using the false discovery rate (FDR) as the threshold value is very strict, we selected *p* < 0.0001 as the threshold value as previously reported [23,24,25,26]. We considered that there is a QTL in the SNP peak place, and in the SNP peak place, there should be three or more three consecutive significant SNPs (*p* < 0.0001) in adjacent significant SNPs with distances less than 270, 150, and 280 kb in the whole population, *indica* subspecies, and *japonica* subspecies, respectively.

## 5. Conclusions

We performed a genome-wide association study (GWAS) in 456 diverse rice core collections from the 3K rice genome. We discovered the nine new QTLs designated as *qSS1-1*, *qSS1-2*, *qSS2-1*, *qSS3-1*, *qSS5-1, qSS5-2*, *qSS7-1*, *qSS8-1*, and *qSS11-1* on chromosome 1, 2, 3, 5, 7, 8, and 11, respectively. We found that the average germination percentage was increased with the number of QTL superior alleles. Then, we explained why *indica* subspecies had superior seed storability to *japonica* subspecies. The Pyramiding of QTL superior alleles of these seed storability-associated genes would be essential for breeding a new rice variety with improved storability.

## Figures and Tables

**Table 1:** The QTLs were detected in the whole population, *indica* subspecies, and *japonica* subspecies, respectively.

QTL Name	Population	Chr.	The Interval of the Significant SNP	Number of the Significant SNP	Lead SNP Position (bp)	-LOG(*P*) of Lead SNP	MAF (%)
*qSS1-2*	Whole population	1	31854852–32063190	26	31937508	6.22	0.27
*qSS2-1*	Whole population	2	22405984–22470838	7	22470838	5.65	0.37
*qSS5-2*	Whole population	5	19789530–20161102	26	20097699	5.66	0.36
*qSS8-1*	Whole population	8	27476715–27509284	9	27479743	5.11	0.33
*qSS2-1*	Indica	2	22405984–22590272	15	22470838	5.93	0.12
*qSS3-1*	Indica	3	9908667–9943096	21	9933469	5.28	0.08
*qSS5-1*	Indica	5	3248113–3450060	19	3369825	4.97	0.08
*qSS5-2*	Indica	5	20007577–20170795	34	20097699	5.33	0.1
*qSS7-1*	Indica	7	13568523–13590543	4	13587640	5.03	0.34
*qSS11-1*	Indica	11	23865348–23902074	3	23902074	5.01	0.14
*qSS1-1*	Japonica	1	12113558–12273000	15	12273000	4.8	0.14

**Table 2:** The lead SNP effect value in the whole population, *indica* subspecies, and *japonica* subspecies, respectively.

Chr.	QTL Name	Position of the Lead SNP	Effect Value (%)		
			Whole Population	*Indica* Subspecies	*Japonica* Subspecies
1	*qSS1-1*	12273000	6.36	0.46	22.97
1	*qSS1-2*	31937508	12.19	9.72	15.30
2	*qSS2-1*	22470838	11.43	12.99	5.62
3	*qSS3-1*	9933469	10.25	32.48	15.70
5	*qSS5-1*	3369825	17.51	24.38	1.71
5	*qSS5-2*	20097699	16.45	17.98	14.31
7	*qSS7-1*	13587640	7.87	8.60	2.05
8	*qSS8-1*	27479743	7.91	6.80	15.61
11	*qSS11-1*	23902074	8.37	10.72	0.33

**Table 3:** The main haplotype analysis of the nine QTLs for seed storability in the whole population (+ superior SNP, − inferior SNP).

Groups	Subspecies	No. of Varieties	No. Superior SNPs	The Average Germination Percentage (%)	Genotype								
					*qSS* *1-1*	*qSS* *1-2*	*qSS* *2-1*	*qSS* *3-1*	*qSS* *5-1*	*qSS* *5-2*	*qSS* *7-1*	*qSS* *8-1*	*qSS* *11-1*
1	*Indica*	1	9	98	+	+	+	+	+	+	+	+	+
2	*Indica*	2	8	92	+	+	+	+	+	-	+	+	+
3	*Indica*	59	7	72.77	+	+	+	-	-	+	+	+	+
4	*Indica*	16	6	77.96	+	+	+	-	-	+	-	+	+
5	*Indica*	49	6	61.74	+	+	+	-	-	+	+	-	+
6	*Indica*	12	5	71.33	-	+	+	-	-	+	+	-	+
7	*Indica*	27	5	60.94	+	+	+	-	-	+	-	-	+
8	*Indica*	7	4	34.76	+	+	+	-	-	+	-	-	-
9	*Japonica*	2	5	91	-	+	+	-	+	-	-	+	+
10	*Japonica*	1	5	80.67	+	+	+	-	-	-	-	+	+
11	*Japonica*	10	4	39.67	+	+	-	-	-	-	-	+	+
12	*Japonica*	31	3	73.12	-	+	-	-	-	-	-	+	+
13	*Japonica*	70	2	15.58	-	-	-	-	-	-	-	+	+
14	*Japonica*	8	1	10.75	-	-	-	-	-	-	-	-	+

## Data Availability

Not applicable.

## Supplementary Materials

The following are available online at https://www.mdpi.com/2223-7747/10/4/812/s1, Table S1. Basic information of 456 rice accessions.
