# Supplementary material for: Genome-Wide Association Study Reveals the QTLs for Seed Storability in World Rice Core Collections
Source: Plants (Basel). 2021 Apr 20;10(4):812. doi: 10.3390/plants10040812 (PMC8074387; doi:10.3390/plants10040812)
Supplement: Supplementary file 1 [file plants-10-00812-s001.zip › plants-1168750-supplementary.pdf]

Table S1. Basic information of 456 rice accessions.

ID	Name	Subpopulation based on PC analysis	K9_group_ Admixture	Genetic_Stock_varname	Country source
C001	IRIS_313-8530	Indica	XI-2	DHANE BURWA::IRGC 10105-1	India
C002	IRIS_313-8935	Indica	XI-adm	ARC 18061::IRGC 47650-1	India
C003	IRIS_313-10327	Japonica	GJ-trp	CAROLINO BLANCO::IRGC 117249-1	Peru
C004	IRIS_313-8768	Japonica	GJ-trp	MOROFIN::IRGC 56698-1	Cote d'Ivoire
C005	IRIS_313-10392	Indica	XI-1B	IR 77390-1-6-4-19-1-B::IRGC 117303-1	Philippines
C006	IRIS_313-11821	Indica	XI-2	19::IRGC 70786-1	India
C007	IRIS_313-11806	Indica	XI-1A	78 XUAN WU::IRGC 70475-1	China
C008	IRIS_313-10509	Indica	XI-3	AMBARIKORI::IRGC 5132-1	West Africa
C009	IRIS_313-11745	Indica	XI-1A	AN FU ZHAN::IRGC 67878-1	China
C010	IRIS_313-11493	Indica	XI-adm	AR 133::IRGC 53942-1	India
C011	IRIS_313-10857	Indica	admix	ARC 10825::IRGC 21082-1	India
C012	IRIS_313-10858	Indica	XI-adm	ARC 10894::IRGC 21122-1	India
C013	IRIS_313-11295	Indica	admix	ARC 11397::IRGC 42664-1	India
C014	IRIS_313-10871	Indica	cA (Aus)	ARC 11777::IRGC 21639-1	India
C015	IRIS_313-10891	Indica	cA (Aus)	ARC 12867::IRGC 22401-1	India
C016	IRIS_313-10892	Indica	cA (Aus)	ARC 12920::IRGC 22450-1	India
C017	IRIS_313-10894	Indica	admix	ARC 13204::IRGC 22558-1	India
C018	IRIS_313-11272	Indica	cA (Aus)	ARC 14756::IRGC 41736-1	India
C019	IRIS_313-11274	Indica	cA (Aus)	ARC 14901::IRGC 41811-1	India
C020	IRIS_313-10666	Indica	XI-adm	ARC 6218::IRGC 12255-1	India
C021	IRIS_313-10852	Indica	cA (Aus)	ARC 7336::IRGC 20606-1	India
C022	IRIS_313-11812	Indica	XI-3	ASFALA::IRGC 70650-1	Kenya
C023	IRIS_313-11048	Indica	cA (Aus)	AUS 171::IRGC 29004-1	Bangladesh
C024	IRIS_313-11050	Indica	cA (Aus)	AUS 233::IRGC 29036-1	Bangladesh
C025	IRIS_313-11051	Indica	cA (Aus)	AUS 242::IRGC 29040-1	Bangladesh
C026	IRIS_313-11055	Indica	cA (Aus)	AUS 299::IRGC 29087-1	Bangladesh
C027	IRIS_313-11057	Indica	cA (Aus)	AUS 308::IRGC 29096-1	Bangladesh
C028	IRIS_313-11058	Indica	cA (Aus)	AUS 329::IRGC 29116-1	Bangladesh
C029	IRIS_313-11656	Indica	XI-adm	B 3913 B 16-20 ST 28::IRGC 63099-1	Indonesia
C030	IRIS_313-11878	Indica	XI-1A	BAI RI XIAN::IRGC 72588-1	China
C031	IRIS_313-11062	Japonica	cB (Bas)	BEGUNBICHI 33::IRGC 29260-1	Bangladesh
C032	IRIS_313-11823	Indica	XI-2	BHU BHUSI::IRGC 70803-1	India
C033	IRIS_313-11788	Japonica	GJ-adm	CANLUBANG::IRGC 69816-1	Philippines
C034	IRIS_313-10997	Indica	XI-3	CEMPO MANGGAR::IRGC 27107-1	Indonesia
C035	IRIS_313-12121	Indica	XI-3	CHA LIENG::IRGC 84920-1	Lao People's Democratic Republic
C036	IRIS_313-11692	Indica	XI-1A	CHIAYI WU-K'O::IRGC 64974-1	Taiwan
C037	IRIS_313-11693	Indica	XI-adm	CHING CH'UNG::IRGC 65002-1	Taiwan
C038	IRIS_313-11737	Indica	cA (Aus)	CHUNDI::IRGC 67486-1	India
C039	IRIS_313-11664	Indica	XI-adm	CUN GU NUO::IRGC 63576-1	China
C040	IRIS_313-11795	Indica	XI-1A	DA GU AI 7::IRGC 70075-1	China
C041	IRIS_313-11720	Indica	XI-3	DAW 85::IRGC 66704-1	Thailand
C042	IRIS_313-11705	Indica	XI-3	DAWK PUD KHAO::IRGC 65808-1	Thailand
C043	IRIS_313-11700	Indica	XI-3	DAW NOK KAEN::IRGC 65548-1	Thailand
C044	IRIS_313-11671	Indica	XI-2	DERAWA::IRGC 64106-1	Nepal
C045	IRIS_313-11824	Indica	XI-2	DHANIYA PHOOL::IRGC 70811-1	India
C046	IRIS_313-11723	Indica	XI-2	DISSIGBE::IRGC 66916-1	Guinea
C047	IRIS_313-10766	Japonica	GJ-trp	DJALAWARA::IRGC 17471-1	Indonesia
C048	IRIS_313-10602	Indica	cA (Aus)	DM 49::IRGC 8775-1	Bangladesh
C049	IRIS_313-12334	Indica	XI-3	DO KHAW::IRGC 106977-1	Lao People's Democratic Republic
C050	IRIS_313-10544	Indica	XI-2	DONGREM::IRGC 6688-1	India

C051	IRIS_313-12193	Indica	XI-3	DO NOUAN::IRGC 89648-1	Lao People's Democratic Republic
C052	IRIS_313-11796	Indica	XI-1A	DU GEN CHUAN::IRGC 70083-1	China
C053	IRIS_313-10605	Indica	cA (Aus)	DV 86::IRGC 8840-1	Bangladesh
C054	IRIS_313-11746	Indica	XI-1A	E 2070::IRGC 67981-1	China
C055	IRIS_313-11747	Indica	XI-adm	E 4197::IRGC 68004-1	China
C056	IRIS_313-12259	Indica	XI-3	EA NOUAN::IRGC 95147-1	Lao People's Democratic Republic
C057	IRIS_313-11798	Indica	XI-1B	E ZI 100::IRGC 70192-1	China
C058	IRIS_313-11799	Indica	XI-1A	E ZI 110::IRGC 70201-1	China
C059	IRIS_313-11801	Indica	XI-adm	FAN WU::IRGC 70245-1	China
C060	IRIS_313-12033	Indica	XI-1A	GAO JIAO HONG::IRGC 80920-1	China
C061	IRIS_313-11748	Indica	XI-adm	GAO JIAO YING GAN ZHAN::IRGC 68053-1	China
C062	IRIS_313-11679	Indica	XI-3	GASET BOW::IRGC 64316-1	Thailand
C063	IRIS_313-11929	Japonica	GJ-trp	GILINGAN MARANGRAS::IRGC 75213-1	Philippines
C064	IRIS_313-10772	Indica	XI-3	GOJOT::IRGC 17669-1	Indonesia
C065	IRIS_313-10503	Indica	XI-1A	HA GOO::IRGC 4577-1	China
C066	IRIS_313-11741	Indica	XI-2	HERATH BANDA::IRGC 67630-1	Sri Lanka
C067	IRIS_313-12128	Indica	XI-3	HMA GNENG::IRGC 85751-1	Lao People's Democratic Republic
C068	IRIS_313-11607	Indica	XI-2	HOLDIGANTHI::IRGC 61105-1	India
C069	IRIS_313-11727	Indica	XI-1A	HONG YANG ZAO 3::IRGC 67177-1	China
C070	IRIS_313-10968	Indica	XI-1A	IH PEN SHIM MING::IRGC 26067-1	Brazil
C071	IRIS_313-12127	Indica	XI-3	INPONG::IRGC 85524-1	Lao People's Democratic Republic
C072	IRIS_313-11979	Indica	XI-1B	IR 19661-364-1-2-3::IRGC 78061-1	Philippines
C073	IRIS_313-11516	Indica	XI-adm	IR 3839-1::IRGC 55946-1	Philippines
C074	IRIS_313-11978	Indica	XI-1B	IR 5657-33-2::IRGC 77992-1	Philippines
C075	IRIS_313-11717	Indica	XI-adm	JATI MANI::IRGC 66565-1	Indonesia
C076	IRIS_313-11802	Indica	XI-1A	JIE CAO ZHAN::IRGC 70305-1	China
C077	IRIS_313-11622	Indica	XI-1A	JUAN YE LAI::IRGC 61825-1	China
C078	IRIS_313-11324	Indica	cA (Aus)	KALABOKRI::IRGC 43872-1	Bangladesh
C079	IRIS_313-11097	Indica	XI-adm	KALAGNON::IRGC 30335-1	Philippines
C080	IRIS_313-11489	Indica	cA (Aus)	KALU T 139::IRGC 53670-1	India
C081	IRIS_313-11816	Indica	XI-adm	KARILETYONE::IRGC 70745-1	Myanmar
C082	IRIS_313-10785	Japonica	GJ-trp	KETAN SLAWI::IRGC 17963-1	Indonesia
C083	IRIS_313-11708	Indica	XI-3	KHAO YAI GUANG::IRGC 65972-1	Thailand
C084	IRIS_313-11814	Indica	XI-3	KIKUBA::IRGC 70722-1	Kenya
C085	IRIS_313-11416	Indica	XI-2	KONAMANI::IRGC 49806-1	India
C086	IRIS_313-11724	Indica	XI-3	KONYAN::IRGC 66931-1	Guinea
C087	IRIS_313-11543	Indica	XI-3	KYUN TAW SEIN::IRGC 58072-1	Myanmar
C088	IRIS_313-11750	Indica	XI-adm	L 10595::IRGC 68109-1	China
C089	IRIS_313-11751	Indica	XI-adm	L 10833::IRGC 68121-1	China
C090	IRIS_313-10999	Japonica	GJ-trp	LANDEO::IRGC 27164-1	Indonesia
C091	IRIS_313-11234	Indica	XI-adm	LIGUNGTUNG::IRGC 38786-1	Philippines
C092	IRIS_313-12048	Indica	XI-3	LIMA::IRGC 81487-1	Indonesia
C093	IRIS_313-10748	Indica	XI-adm	LUA DUC::IRGC 16718-1	Viet Nam
C094	IRIS_313-11667	Indica	XI-1A	LUO AI ZAO 3::IRGC 63730-1	China
C095	IRIS_313-11728	Indica	XI-3	LUO SI ZHAN::IRGC 67211-1	China
C096	IRIS_313-11752	Indica	XI-1A	M 6034-1::IRGC 68193-1	China
C097	IRIS_313-12349	Japonica	GJ-sbtrp	MAK KHEUA DENG::IRGC 107709-1	Lao People's Democratic Republic
C098	IRIS_313-12350	Japonica	GJ-sbtrp	MAK KHEUA KANG::IRGC 107712-1	Lao People's Democratic Republic
C099	IRIS_313-11736	Japonica	GJ-trp	MALAGKIT (PINELIPE)::IRGC 67444-1	Philippines
C100	IRIS_313-11945	Indica	XI-2	MALIKULI::IRGC 76237-1	Bangladesh

C101	IRIS_313-11882	Indica	XI-1A	MAN TIAN QING::IRGC 72776-1	China
C102	IRIS_313-12066	Indica	XI-3	MARAMAY::IRGC 82628-1	Philippines
C103	IRIS_313-10609	Indica	XI-adm	MATHOLUWA::IRGC 8901-1	Sri Lanka
C104	IRIS_313-11784	Indica	XI-1B	MBEIMBEIHUN::IRGC 69730-1	Sierra Leone
C105	IRIS_313-11760	Indica	XI-2	MENAHARA 505::IRGC 68709-1	Madagascar
C106	IRIS_313-10794	Japonica	GJ-trp	MENDUNGAN::IRGC 18245-1	Indonesia
C107	IRIS_313-11811	Indica	XI-2	MOSHI::IRGC 70648-1	Kenya
C108	IRIS_313-11815	Indica	XI-3	MOSHI::IRGC 70730-1	Kenya
C109	IRIS_313-10440	Japonica	GJ-tmp	NAGKAYAT::IRGC 584-1	Philippines
C110	IRIS_313-12355	Indica	XI-adm	NASAENG::IRGC 107819-1	Lao People's Democratic Republic
C111	IRIS_313-11642	Indica	XI-2	NCS 599::IRGC 62373-1	India
C112	IRIS_313-11643	Indica	XI-1B	NCS 603 B::IRGC 62377-1	India
C113	IRIS_313-11644	Indica	XI-2	NCS 745::IRGC 62466-1	India
C114	IRIS_313-11645	Indica	XI-2	NCS 766::IRGC 62478-1	India
C115	IRIS_313-11648	Indica	XI-2	NCS 830::IRGC 62518-1	India
C116	IRIS_313-11683	Indica	XI-3	NIAW KHIAW NGOO::IRGC 64563-1	Thailand
C117	IRIS_313-11841	Indica	XI-3	NIAW NARAI::IRGC 71353-1	Thailand
C118	IRIS_313-11762	Indica	XI-2	NS 1515::IRGC 68947-1	Madagascar
C119	IRIS_313-11761	Indica	XI-2	NS 199::IRGC 68864-1	Cote d'Ivoire
C120	IRIS_313-11764	Indica	XI-3	P 41::IRGC 68987-1	Liberia
C121	IRIS_313-10954	Indica	XI-3	PADI GRUNDUL 1::IRGC 25493-1	Indonesia
C122	IRIS_313-10682	Indica	XI-3	PA KHENG::IRGC 12996-1	Lao People's Democratic Republic
C123	IRIS_313-11820	Indica	XI-3	PAKISTANHMWE::IRGC 70763-1	Myanmar
C124	IRIS_313-11738	Indica	XI-2	PAUNDRI::IRGC 67527-1	India
C125	IRIS_313-12135	Indica	XI-1B	PERUBAK LUEY::IRGC 86436-1	Malaysia
C126	IRIS_313-11763	Indica	XI-adm	PJ 110::IRGC 68981-1	Cameroon
C127	IRIS_313-11685	Indica	XI-3	PLAWNG SAENG::IRGC 64595-1	Thailand
C128	IRIS_313-11730	Indica	XI-adm	QING ER XIAO 2::IRGC 67255-1	China
C129	IRIS_313-11731	Indica	XI-adm	QING TAI AI::IRGC 67273-1	China
C130	IRIS_313-11732	Indica	XI-1A	QING ZAO 3::IRGC 67281-1	China
C131	IRIS_313-11807	Indica	XI-adm	R 582::IRGC 70504-1	Colombia
C132	IRIS_313-10649	Japonica	GJ-trp	RACION 1::IRGC 11340-1	Philippines
C133	IRIS_313-11191	Indica	cA (Aus)	RANRUWAN::IRGC 36360-1	Sri Lanka
C134	IRIS_313-11668	Indica	XI-1A	RONG DAO 4::IRGC 63820-1	China
C135	IRIS_313-11789	Indica	XI-2	SABORAMANDALOAFO::IRGC 69875-1	Madagascar
C136	IRIS_313-11436	Japonica	GJ-trp	SAKAZELE (538)::IRGC 50880-1	Cote d'Ivoire
C137	IRIS_313-11034	Indica	cA (Aus)	SANTHI 206::IRGC 28215-1	Pakistan
C138	IRIS_313-11460	Indica	XI-2	SELHI::IRGC 52760-1	India
C139	IRIS_313-11733	Indica	XI-1A	SHUANG BAI AI 2::IRGC 67309-1	China
C140	IRIS_313-11734	Indica	XI-1A	SI CHAO 1::IRGC 67335-1	China
C141	IRIS_313-10941	Indica	XI-adm	SINTHA::IRGC 24687-1	Indonesia
C142	IRIS_313-11655	Japonica	GJ-tmp	SI WAN 14::IRGC 63019-1	China
C143	IRIS_313-11716	Indica	XI-3	SOSSOKA::IRGC 66478-1	Guinea
C144	IRIS_313-11089	Indica	XI-3	SRAU THMOR::IRGC 29904-1	Cambodia
C145	IRIS_313-11787	Indica	XI-2	TANDAKAY FINGO::IRGC 69793-1	Gambia
C146	IRIS_313-12024	Indica	XI-1B	TOS 9795::IRGC 80142-1	Sierra Leone
C147	IRIS_313-11794	Indica	XI-2	TSIPALA MENAHAR::IRGC 69979-1	Madagascar
C148	IRIS_313-10603	Indica	cA (Aus)	UCP 122::IRGC 8794-1	Bangladesh
C149	IRIS_313-11740	Indica	XI-adm	UGAGA::IRGC 67604-1	Ghana
C150	IRIS_313-11791	Indica	XI-2	VARY LAHY::IRGC 69908-1	Madagascar
C151	IRIS_313-11753	Indica	XI-1A	XIANG CHANG ZAO::IRGC 68268-1	China
C152	IRIS_313-11805	Indica	XI-1A	XI HONG GU::IRGC 70416-1	China
C153	IRIS_313-11453	Indica	XI-2	ZINYA KOLAMBA::IRGC 52402-1	India
C154	IRIS_313-11528	Indica	XI-3	ZO::IRGC 56914-1	Cote d'Ivoire
C155	IRIS_313-11202	Japonica	GJ-tmp	4583::IRGC 36894-2	China

C156	IRIS_313-10859	Indica	XI-adm	ARC 10939::IRGC 21150-2	India
C157	IRIS_313-10863	Indica	XI-2	ARC 11322::IRGC 21315-2	India
C158	IRIS_313-11256	Indica	XI-adm	ARC 12411::IRGC 41047-2	India
C159	IRIS_313-10888	Japonica	GJ-sbtrp	ARC 12726::IRGC 22288-2	India
C160	IRIS_313-11257	Indica	XI-adm	ARC 12757::IRGC 41095-2	India
C161	IRIS_313-11297	Japonica	admix	ARC 13460::IRGC 42732-2	India
C162	IRIS_313-11258	Japonica	cB (Bas)	ARC 13502::IRGC 41126-2	India
C163	IRIS_313-11260	Indica	XI-adm	ARC 13591::IRGC 41177-2	India
C164	IRIS_313-11262	Indica	XI-adm	ARC 13888::IRGC 41288-2	India
C165	IRIS_313-11263	Indica	XI-adm	ARC 13919::IRGC 41313-2	India
C166	IRIS_313-11266	Indica	XI-adm	ARC 14347::IRGC 41517-2	India
C167	IRIS_313-11267	Indica	XI-2	ARC 14358::IRGC 41523-2	India
C168	IRIS_313-11269	Indica	XI-adm	ARC 14632::IRGC 41650-2	India
C169	IRIS_313-11273	Indica	XI-adm	ARC 14860::IRGC 41793-2	India
C170	IRIS_313-11301	Indica	XI-2	ARC 14868::IRGC 43009-2	India
C171	IRIS_313-11289	Japonica	cB (Bas)	ARC 18578::IRGC 42459-2	India
C172	IRIS_313-10847	Indica	XI-adm	ARC 6015::IRGC 20314-2	India
C173	IRIS_313-12109	Indica	XI-3	ARITH::IRGC 84347-2	Cambodia
C174	IRIS_313-10995	Indica	XI-3	ASE BAKKA LOMPO::IRGC 26988-2	Indonesia
C175	IRIS_313-11047	Indica	cA (Aus)	AUS 84::IRGC 28947-2	Bangladesh
C176	IRIS_313-11239	Indica	XI-adm	B 737 G-KN-23-1::IRGC 39203-2	Indonesia
C177	IRIS_313-10576	Indica	XI-2	BAANYALOJOPOIHUN::IRGC 7928-2	Sierra Leone
C178	IRIS_313-11350	Japonica	cB (Bas)	BAJAL::IRGC 45024-2	India
C179	IRIS_313-11351	Indica	XI-2	BANCHI::IRGC 45055-2	India
C180	IRIS_313-10942	Indica	XI-3	BANDANG BUNGKUAKLAN::IRGC 24697-2	Indonesia
C181	IRIS_313-11241	Indica	XI-2	BR 116-3B-53::IRGC 39559-2	Bangladesh
C182	IRIS_313-10765	Japonica	GJ-trp	CERE KAWAT::IRGC 17401-2	Indonesia
C183	IRIS_313-11240	Indica	XI-adm	CR 157-392-4::IRGC 39247-2	India
C184	IRIS_313-10986	Indica	XI-2	HALDI JAON::IRGC 26638-2	Bangladesh
C185	IRIS_313-10614	Indica	XI-adm	HAM MOON::IRGC 9135-2	Hong Kong
C186	IRIS_313-10824	Indica	XI-3	HOING CANTEL::IRGC 19282-2	Indonesia
C187	IRIS_313-10718	Indica	cA (Aus)	KARUTHA SEENATI::IRGC 15515-2	Sri Lanka
C188	IRIS_313-11205	Indica	XI-2	KAZALSAIL::IRGC 37161-2	Bangladesh
C189	IRIS_313-11386	Indica	XI-3	KHAO DAWK MALI TIA::IRGC 47773-2	Thailand
C190	IRIS_313-11817	Indica	XI-adm	KHAOSAING::IRGC 70746-2	Myanmar
C191	IRIS_313-11039	Indica	XI-1A	KWANG LU AI 4::IRGC 28480-2	China
C192	IRIS_313-11338	Indica	XI-adm	LOBANG (WHITE)::IRGC 44548-2	Philippines
C193	IRIS_313-10755	Indica	XI-3	NEP BA BONG TO::IRGC 17005-2	Viet Nam
C194	IRIS_313-11242	Indica	XI-2	OR 117-8::IRGC 39680-2	India
C195	IRIS_313-11224	Indica	XI-2	PATNAI 31-679::IRGC 38149-2	Bangladesh
C196	IRIS_313-11079	Indica	XI-3	PHAN PHAE::IRGC 29652-2	Lao People's Democratic Republic
C197	IRIS_313-11245	Indica	XI-adm	RP 20-12::IRGC 40031-2	India
C198	IRIS_313-11228	Indica	XI-2	SADAJIRA 19-339::IRGC 38361-2	Bangladesh
C199	IRIS_313-11392	Indica	XI-3	SA MER::IRGC 48454-2	Thailand
C200	IRIS_313-10814	Indica	XI-3	SIBORAS::IRGC 18816-2	Indonesia
C201	IRIS_313-10816	Japonica	GJ-trp	SIDJERO GUNDIL::IRGC 18829-2	Indonesia
C202	IRIS_313-11229	Indica	XI-2	STRAW 23-428::IRGC 38416-2	Bangladesh
C203	IRIS_313-11372	Indica	XI-2	SUFALDHULA::IRGC 46698-2	India
C204	IRIS_313-11081	Indica	XI-3	THAO LOUANG::IRGC 29688-2	Lao People's Democratic Republic
C205	IRIS_313-10820	Indica	XI-3	TJERE MANGGA::IRGC 19116-2	Indonesia
C206	IRIS_313-11037	Indica	cA (Aus)	421::IRGC 28458-1	Pakistan
C207	IRIS_313-12252	Japonica	GJ-sbtrp	A MOUK::IRGC 94474-1	Lao People's Democratic Republic
C208	IRIS_313-11495	Japonica	GJ-trp	ANENOE::IRGC 54140-1	Indonesia

C209	IRIS_313-10883	Japonica	admix	ARC 12490::IRGC 22095-1	India
C210	IRIS_313-10708	Japonica	GJ-trp	B 6311 A 5553-16-2::IRGC 14541-1	United States of America
C211	IRIS_313-11834	Japonica	GJ-sbtrp	BUENG MONG LENG WE::IRGC 71088-1	Thailand
C212	IRIS_313-11673	Japonica	GJ-trp	BURI BURING::IRGC 64174-1	Philippines
C213	IRIS_313-10570	Japonica	GJ-tmp	CHUSEI HONEN::IRGC 7777-1	Japan
C214	IRIS_313-11689	Japonica	GJ-tmp	DECHANGBYEO::IRGC 64858-1	Korea, Republic of
C215	IRIS_313-10677	Japonica	GJ-tmp	DEWAMINORI::IRGC 12743-1	Japan
C216	IRIS_313-12258	Japonica	GJ-sbtrp	DON::IRGC 95117-1	Lao People's Democratic Republic
C217	IRIS_313-11677	Indica	XI-3	E NAWN KHAO::IRGC 64293-1	Thailand
C218	IRIS_313-11835	Indica	XI-adm	E NOI NAH::IRGC 71138-1	Thailand
C219	IRIS_313-11651	Japonica	GJ-tmp	FEI ZHAO 12::IRGC 62683-1	China
C220	IRIS_313-12018	Japonica	GJ-trp	FRENSI GBOI::IRGC 79983-1	Sierra Leone
C221	IRIS_313-11635	Indica	XI-3	GAM PERNG::IRGC 62135-1	Thailand
C222	IRIS_313-11652	Japonica	GJ-tmp	GONG SHE 9::IRGC 62693-1	China
C223	IRIS_313-12054	Japonica	GJ-tmp	HUA 24::IRGC 82127-1	China
C224	IRIS_313-10834	Japonica	GJ-sbtrp	IARI 11387::IRGC 19573-1	India
C225	IRIS_313-10828	Japonica	GJ-trp	IKOGAN::IRGC 19412-1	Philippines
C226	IRIS_313-11722	Indica	XI-adm	KABERI::IRGC 66801-1	Bangladesh
C227	IRIS_313-10681	Indica	XI-3	KHAN THAI DO::IRGC 12990-1	Lao People's Democratic Republic
C228	IRIS_313-10928	Indica	XI-3	KHAO' SIM::IRGC 24094-1	Thailand
C229	IRIS_313-12060	Japonica	GJ-adm	LIJIAN 942::IRGC 82399-1	China
C230	IRIS_313-11467	Indica	XI-3	MACAPAGAL::IRGC 52927-1	Philippines
C231	IRIS_313-11658	Japonica	GJ-trp	MANYALOJOPOIHUN::IRGC 63350-1	Sierra Leone
C232	IRIS_313-11663	Indica	admix	MARHARORA::IRGC 63511-1	Zimbabwe
C233	IRIS_313-11653	Japonica	GJ-tmp	MA SHE 8::IRGC 62750-1	China
C234	IRIS_313-11809	Indica	cA (Aus)	MHARAKA::IRGC 70612-1	Kenya
C235	IRIS_313-12129	Japonica	GJ-sbtrp	MON LAI::IRGC 85857-1	Lao People's Democratic Republic
C236	IRIS_313-11435	Japonica	GJ-trp	MONMINEUPLEU::IRGC 50873-1	Cote d'Ivoire
C237	IRIS_313-11739	Japonica	GJ-trp	NAKPUI::IRGC 67594-1	Ghana
C238	IRIS_313-11924	Japonica	GJ-sbtrp	NAM JAM::IRGC 75069-1	Thailand
C239	IRIS_313-12071	Japonica	GJ-sbtrp	NIA::IRGC 82728-1	Lao People's Democratic Republic
C240	IRIS_313-11786	Indica	XI-3	N'YA NOFF::IRGC 69749-1	Gambia
C241	IRIS_313-11189	Japonica	admix	PAIZ AMBARBU::IRGC 36179-1	Union of Soviet Socialist Republics (Former)
C242	IRIS_313-11900	Japonica	GJ-sbtrp	PLE LIA::IRGC 73562-1	Thailand
C243	IRIS_313-10582	Japonica	GJ-trp	QINOGNAS::IRGC 8135-1	Philippines
C244	IRIS_313-11804	Indica	XI-1A	QUN XUAN ZAO::IRGC 70371-1	China
C245	IRIS_313-11033	Japonica	admix	RATRIO::IRGC 28181-1	Pakistan
C246	IRIS_313-12281	Japonica	GJ-trp	SALAZANA::IRGC 97219-1	Madagascar
C247	IRIS_313-11691	Japonica	GJ-sbtrp	SHANGYIPA::IRGC 64928-1	Bhutan
C248	IRIS_313-12228	Japonica	GJ-sbtrp	SOM::IRGC 92221-1	Lao People's Democratic Republic
C249	IRIS_313-12312	Japonica	GJ-sbtrp	TA SENG::IRGC 99176-1	Lao People's Democratic Republic
C250	IRIS_313-11617	Indica	cA (Aus)	UPRH 33::IRGC 61505-1	India
C251	IRIS_313-11767	Indica	admix	VARY LAVA DE BOLOGNE::IRGC 69101-1	Madagascar
C252	IRIS_313-11792	Japonica	GJ-trp	VONBOANGY::IRGC 69947-1	Madagascar
C253	IRIS_313-10840	Japonica	GJ-tmp	YE ZO::IRGC 19888-1	Korea, Republic of
C254	IRIS_313-11035	Indica	admix	ZIRA SHAHI::IRGC 28327-1	Pakistan
C255	IRIS_313-11725	Japonica	GJ-tmp	ZUIHOU::IRGC 66982-1	Japan

C256	IRIS_313-10725	Indica	XI-adm	ERNEST::IRGC 15884-2	Senegal
C257	IRIS_313-10779	Indica	XI-3	KERAN DUKUH::IRGC 17822-2	Indonesia
C258	IRIS_313-11077	Japonica	GJ-sbtrp	LAI HANG HMA::IRGC 29608-2	Lao People's Democratic Republic
C259	IRIS_313-8349	Indica	XI-2	LATA MONA::IRGC 49333-1	Bangladesh
C260	B001	Japonica	GJ-tmp	Heibiao	China
C261	B003	Japonica	GJ-adm	Zaoshengbai	China
C262	B008	Japonica	GJ-tmp	Baxiang	Viet Nam
C263	B011	Indica	XI-adm	CO 22	India
C264	B014	Japonica	GJ-tmp	Wuziluosi 215	Uzbekistan
C265	B015	Indica	XI-1A	Kahamu	Romania
C266	B016	Japonica	GJ-tmp	Aomierte 168	Hungary
C267	B018	Japonica	GJ-trp	American Huangkedao	United States of America
C268	B023	Japonica	GJ-tmp	Gaoliqiu	Democratic People's Republic of Korea
C269	B026	Indica	XI-adm	Padi Ladang Ase Polo Komek	Indonesia
C270	B027	Indica	XI-3	C 894-21	Philippines
C271	B038	Japonica	GJ-tmp	Nabated A Smar	Egypt
C272	B040	Indica	XI-adm	K 24	India
C273	B045	Japonica	GJ-tmp	Gongchengxiang	Japan
C274	B046	Japonica	GJ-tmp	Qiutianxiaoting	Japan
C275	B183	Japonica	GJ-tmp	Qingnuo Kyohatamochi	Japan
C276	B190	Japonica	GJ-trp	ITA 221	Nigeria
C277	B053	Japonica	GJ-trp	80 A 97 YR 303-304-1-3	Australia
C278	B197	Indica	XI-1A	Taizhongxianxuan 2	Taiwan
C279	B204	Japonica	GJ-tmp	Longhuamaohu	China
C280	B070	Japonica	GJ-adm	Laoguangtou 83	China
C281	B081	Indica	XI-1A	Heidu 4	China
C282	B212	Japonica	GJ-tmp	Chikenuo	China
C283	B214	Indica	XI-1B	Xuanenchangtanqingzhan	China
C284	B216	Indica	XI-1A	Hanmadao4	China
C285	B090	Indica	XI-adm	Zimi	China
C286	B218	Japonica	GJ-tmp	Wuzidui	China
C287	B094	Indica	XI-1A	Zinuo	China
C288	B097	Indica	XI-adm	Jiienuo	China
C289	B221	Indica	XI-1A	Fanhaopi	China
C290	B100	Japonica	GJ-tmp	Hongkezhenuo	China
C291	B225	Japonica	GJ-tmp	Guantuibaihe 1	China
C292	B229	Indica	XI-adm	Menjiading 2	China
C293	B111	Japonica	GJ-tmp	Zimangfeie	China
C294	B116	Indica	XI-adm	Shufeng 101	China
C295	B125	Indica	XI-adm	Xiangwanxian 3	China
C296	B129	Indica	XI-adm	Hongainuo	China
C297	B149	Indica	XI-1A	Haoxiang	China
C298	B247	Indica	XI-adm	Jinnante B	China
C299	B261	Indica	XI-1A	Meihuanuo	China
C300	B160	Japonica	GJ-tmp	Shuiyuan 300Li	China
C301	B266	Japonica	GJ-sbtrp	Haomake (K)	China
C302	B164	Indica	cA (Aus)	Qingke	China
C303	IRIS_313-9422	Indica	cA (Aus)	CHUNGUR BALI::IRGC 25855-1	Bangladesh
C304	IRIS_313-10397	Indica	XI-1B	IRGA 318-11-6-9-2B::IRGC 117339-1	Colombia
C305	IRIS_313-8341	Indica	XI-adm	BAT DO::IRGC 7014-1	Viet Nam
C306	IRIS_313-8173	Japonica	GJ-trp	A 201::GERVEX 1638-C1	United States of America
C307	IRIS_313-7638	Indica	XI-adm	ANGIFOTSY 685::GERVEX 4717-C1	Madagascar
C308	IRIS_313-8037	Japonica	GJ-adm	DORELLA::GERVEX 100-C1	Italy

C309	IRIS_313-8172	Japonica	admix	FIDJI::GERVEX 1636-C1	Philippines
C310	IRIS_313-8185	Japonica	GJ-trp	GLADIO::GERVEX 1652-C1	Italy
C311	IRIS_313-8074	Japonica	GJ-trp	GOOLARAH::GERVEX 500-C1	Australia
C312	IRIS_313-8075	Japonica	GJ-tmp	HARRA::GERVEX 501-C1	Australia
C313	IRIS_313-7689	Indica	XI-1B	IR 2307-247-2-2-3::IRGC 77982-C1	Philippines
C314	IRIS_313-7902	Japonica	GJ-trp	IR 63380-16::C1	Philippines
C315	IRIS_313-8010	Japonica	GJ-trp	IR 71524-44-1-1::C1	Philippines
C316	IRIS_313-7909	Japonica	GJ-trp	IR 71525-19-1-1::C1	Philippines
C317	IRIS_313-7914	Japonica	GJ-trp	IRAT 112::C1	Cote d'Ivoire
C318	IRIS_313-8123	Japonica	GJ-tmp	MUGA::GERVEX 1099-C1	Portugal
C319	IRIS_313-8024	Japonica	GJ-tmp	RIBE 253::GERVEX 54-C1	Italy
C320	IRIS_313-7994	Japonica	GJ-trp	VARY MADINIKA 3566::GERVEX 8448-C1	Madagascar
C321	IRIS_313-7808	Indica	XI-1B	WAS 173-B-B-6-2-2::C1	Senegal
C322	IRIS_313-8167	Japonica	GJ-adm	DELTA::GERVEX 1519-C1	France
C323	IRIS_313-7646	Japonica	admix	BETSILAIZINA::GERVEX 8405-C1	Madagascar
C324	IRIS_313-8454	Indica	XI-1A	LAI YIP ZIM::IRGC 4955-1	Taiwan
C325	IRIS_313-8502	Japonica	GJ-tmp	M 102::IRGC 76307-1	United States of America
C326	IRIS_313-8434	Japonica	GJ-trp	TEBONNET::IRGC 66760-1	United States of America
C327	IRIS_313-8697	Indica	XI-3	YEBAWYIN::IRGC 33885-1	Myanmar
C328	IRIS_313-8986	Indica	XI-adm	ARC 10754::IRGC 12603-1	India
C329	IRIS_313-8967	Indica	XI-adm	ARC 12576::IRGC 22163-1	India
C330	IRIS_313-8982	Indica	XI-adm	ARC 18112::IRGC 42274-1	India
C331	IRIS_313-9066	Indica	XI-1B	AUS 177::IRGC 29009-1	Bangladesh
C332	IRIS_313-9148	Indica	XI-2	BADAL 1163::IRGC 32796-1	Bangladesh
C333	IRIS_313-8923	Japonica	GJ-trp	CALIFORNIA BELLE::IRGC 66836-1	United States of America
C334	IRIS_313-9966	Indica	XI-1B	CICA 9::IRGC 53079-1	Colombia
C335	IRIS_313-9023	Indica	XI-adm	CR 60-10::IRGC 15777-1	India
C336	IRIS_313-10177	Indica	XI-adm	DA GANG ZHAN::IRGC 67103-1	China
C337	IRIS_313-10109	Indica	XI-3	EX EBOKOZURU::IRGC 79912-1	Nigeria
C338	IRIS_313-10114	Indica	XI-2	FACAGRO 64::IRGC 82059-1	Burundi
C339	IRIS_313-8909	Indica	XI-3	FAYA MOSHI::IRGC 69653-1	Tanzania
C340	IRIS_313-9302	Indica	XI-3	GAM PAI 30-12-15::IRGC 831-1	Thailand
C341	IRIS_313-9472	Indica	XI-adm	H 6::IRGC 157-1	Sri Lanka
C342	IRIS_313-10080	Japonica	GJ-trp	HIRAKAWA OKUTE::IRGC 74554-1	Japan
C343	IRIS_313-9602	Indica	XI-adm	HTA 22::IRGC 45827-1	Thailand
C344	IRIS_313-9922	Indica	XI-1B	IRI 339::IRGC 46956-1	Korea, Republic of
C345	IRIS_313-9262	Indica	XI-2	JHODI BIRUN::IRGC 31812-1	Bangladesh
C346	IRIS_313-9590	Indica	XI-3	KETAN SERANG::IRGC 14615-1	Indonesia
C347	IRIS_313-8925	Indica	XI-2	KURULUTUDU::IRGC 36304-1	Sri Lanka
C348	IRIS_313-9112	Indica	XI-3	LEUANG YAI 344::IRGC 651-1	Thailand
C349	IRIS_313-10097	Japonica	GJ-tmp	MAEKJO::IRGC 77666-1	Korea, Republic of
C350	IRIS_313-8930	Indica	XI-1B	MUKKALA BAZAL::IRGC 77279-1	Bangladesh
C351	IRIS_313-10134	Indica	XI-3	PIN GAEW 56::IRGC 7887-1	Thailand
C352	IRIS_313-10235	Indica	XI-adm	PSBRC 68::IRGC 99711-1	Philippines
C353	IRIS_313-10078	Japonica	GJ-trp	YOSHINO MOCHI::IRGC 74550-1	Japan
C354	IRIS_313-10333	Indica	XI-1B	B 6136-3-TB-0-1-5::IRGC 117312-1	Indonesia
C355	IRIS_313-9184	Indica	XI-adm	XI GAN JING REN::IRGC 60035-1	China
C356	IRIS_313-8453	Indica	XI-adm	ARC 18597::IRGC 43299-1	India
C357	IRIS_313-10301	Indica	XI-adm	IRGA 959-1-2-2F-4-1-4A-6-CA-6X::IRGC 117006-1	Brazil
C358	IRIS_313-8586	Indica	XI-3	PLI KHAO::IRGC 64596-1	Thailand
C359	IRIS_313-8755	Japonica	GJ-tmp	NORIN 6::IRGC 2633-1	Japan
C360	IRIS_313-9297	Japonica	GJ-trp	KORR::IRGC 30751-1	Liberia

C361	IRIS_313-8356	Japonica	GJ-trp	BLICCA::IRGC 47151-1	Philippines
C362	IRIS_313-8658	Japonica	GJ-adm	CP 231::IRGC 34018-1	United States of America
C363	IRIS_313-8751	Indica	XI-3	HNANWA::IRGC 33118-1	Myanmar
C364	IRIS_313-8595	Indica	XI-2	MAKALIOKA STANDARD::IRGC 12768-1	Madagascar
C365	IRIS_313-8725	Indica	XI-3	PULUT BARAYA::IRGC 27393-1	Indonesia
C366	IRIS_313-8305	Indica	XI-2	URAIBOOL::IRGC 52785-1	India
C367	CX2	Indica	XI-adm	BR 11	Bangladesh
C368	CX4	Japonica	XI-adm	93072	China
C369	CX6	Japonica	admix	C418	China
C370	CX10	Indica	XI-1A	Gang 46B	China
C371	CX11	Japonica	GJ-adm	Gumei 2	China
C372	CX16	Japonica	GJ-tmp	Yuan jing 7	China
C373	CX59	Japonica	cB (Bas)	Milagrosa, Zawa Banday	Philippines
C374	CX64	Indica	XI-3	IRAT 352	Brazil
C375	CX72	Japonica	cB (Bas)	Basmati 385	Pakistan
C376	CX80	Indica	XI-1B	OM 1706	Viet Nam
C377	CX82	Indica	XI-1B	X 21	Viet Nam
C378	CX84	Indica	XI-adm	X 23	Viet Nam
C379	CX89	Indica	XI-adm	M 202	United States of America
C380	CX90	Indica	XI-1B	Chhomromg	Nepal
C381	CX97	Indica	XI-1B	Budda	India
C382	CX98	Indica	XI-2	Doddabyranella	India
C383	CX99	Indica	XI-2	Doddi	India
C384	CX101	Indica	XI-1A	Hei Mi Chan	China
C385	CX102	Indica	XI-1A	Tek Si Chut	Taiwan
C386	CX103	Indica	admix	Rubio	Peru
C387	CX106	Japonica	GJ-trp	SAL BUI BAO	Viet Nam
C388	CX107	Indica	XI-3	Tun Sart	Viet Nam
C389	CX109	Japonica	GJ-adm	Dacca 6	India
C390	CX111	Japonica	GJ-trp	Giza 14	Egypt
C391	CX112	Japonica	admix	Ginga	Japan
C392	CX114	Indica	XI-1A	Guang Lu Ai 4	China
C393	CX117	Indica	XI-adm	IR 65600-27-1-2-2	Philippines
C394	CX118	Indica	XI-adm	Yetuozai	China
C395	CX120	Indica	XI-adm	TKM 6	India
C396	CX121	Indica	XI-1B	Guang122	China
C397	CX124	Indica	XI-adm	IR 71466-75-3-B-1	Philippines
C398	CX125	Indica	XI-1B	CAOZHAO-2	China
C399	CX134	Indica	XI-1B	IRBB 7	Philippines
C400	CX138	Japonica	GJ-adm	Qd_441	China
C401	CX139	Japonica	GJ-adm	Amareles	Portugal
C402	CX142	Japonica	GJ-adm	Ai Yeh Lu	China
C403	CX143	Japonica	cB (Bas)	Khasar	Iran
C404	CX147	Indica	XI-adm	Seberang	Malaysia
C405	CX149	Japonica	cB (Bas)	Karnal Local	India
C406	CX150	Indica	XI-1B	Chorofa	Philippines
C407	CX152	Indica	XI-1B	Cisadane	Indonesia
C408	CX155	Indica	XI-2	Madhukar	India
C409	CX158	Indica	XI-3	Shwe War Tun	Myanmar
C410	CX160	Indica	XI-2	W 1263	India
C411	CX162	Indica	XI-1A	TN1	Taiwan
C412	CX182	Indica	XI-adm	SLG 1	Japan
C413	CX206	Indica	XI-1B	IR 2061-522-6-9	Philippines
C414	CX207	Indica	XI-adm	Gaozi	China

C415	CX219	Indica	XI-adm	FL 478	Philippines
C416	CX220	Japonica	GJ-trp	IRAT 109	Cote d'Ivoire
C417	CX225	Indica	XI-1B	IR 77298-14-1-2	Philippines
C418	CX226	Indica	XI-1B	IR 06G113	Philippines
C419	CX227	Indica	cA (Aus)	KASALATH	India
C420	CX230	Indica	XI-1B	IR 64-IL	Philippines
C421	CX235	Indica	XI-adm	NSIC RC 9	Philippines
C422	CX238	Indica	XI-1B	BW 311-9	Sri Lanka
C423	CX241	Japonica	GJ-trp	IAC 47	Brazil
C424	CX262	Japonica	GJ-trp	IAC 3	Brazil
C425	CX263	Indica	XI-adm	PADISENEMOK	Malaysia
C426	CX269	Japonica	GJ-trp	IAC 165	Brazil
C427	CX270	Indica	XI-1A	TAICHUNGNATIVE1	Taiwan
C428	CX280	Japonica	admix	Maravilha	Brazil
C429	CX284	Japonica	GJ-adm	Han 502	China
C430	CX285	Japonica	GJ-trp	Nonglinnuo	China
C431	CX286	Japonica	admix	C 349	China
C432	CX288	Indica	XI-adm	D 11	India
C433	CX296	Indica	XI-adm	IR 74	Philippines
C434	CX303	Indica	XI-adm	LX 2007	China
C435	CX305	Indica	XI-1B	ZH 5	China
C436	CX313	Indica	XI-adm	R106	China
C437	CX314	Indica	XI-1B	117	China
C438	CX317	Japonica	GJ-tmp	MR 19	Malaysia
C439	CX342	Indica	XI-adm	Wanxian 763	China
C440	CX344	Japonica	GJ-tmp	Hexi 41	China
C441	CX353	Japonica	GJ-adm	CAU 1	Viet Nam
C442	CX355	Japonica	GJ-trp	Yunlu 102	China
C443	CX356	Japonica	GJ-tmp	Yandao 8	China
C444	CX357	Indica	XI-1B	PSBRC 80	Philippines
C445	CX359	Japonica	GJ-trp	Yunlu 103	China
C446	CX364	Indica	XI-1B	42686	_no_info
C447	CX366	Indica	XI-adm	Ganwanxian 37 (926)	China
C448	CX367	Japonica	GJ-trp	Haogelao	China
C449	CX374	Japonica	GJ-trp	NERICA 9	Cote d'Ivoire
C450	CX380	Japonica	GJ-tmp	Yandao 9	China
C451	CX383	Japonica	GJ-tmp	Hongza	China
C452	CX385	Indica	XI-1B	NPT-100	China
C453	CX389	Japonica	GJ-tmp	Jinyuan 85	China
C454	CX397	Japonica	GJ-tmp	Nangeng 46 (Soft Rice)	China
C455	CX542	Indica	XI-adm	RR2-6	China
C456	IRIS_313-10642	Japonica	GJ-tmp	SACHIKAZE::IRGC 10891-1	Japan
